# A Three-Gene Expression Signature Identifies a Cluster of Patients with Short Survival in Chronic Lymphocytic Leukemia

**DOI:** 10.1155/2019/9453539

**Published:** 2019-11-07

**Authors:** Adrián Mosquera Orgueira, Beatriz Antelo Rodríguez, José Ángel Díaz Arias, Nicolás Díaz Varela, José Luis Bello López

**Affiliations:** ^1^Health Research Institute of Santiago de Compostela (IDIS), Santiago, Spain; ^2^Complexo Hospitalario Universitario de Santiago de Compostela (CHUS), Division of Hematology, SERGAS, Santiago, Spain; ^3^University of Santiago de Compostela, Santiago, Spain

## Abstract

Chronic lymphocytic leukemia (CLL) is a lymphoproliferative disorder characterized by its heterogeneous clinical evolution. Despite the discovery of the most frequent cytogenomic drivers of disease during the last decade, new efforts are needed in order to improve prognostication. In this study, we used gene expression data of CLL samples in order to discover novel transcriptomic patterns associated with patient survival. We observed that a 3-gene expression signature composed of *SCGB2A1*, *KLF4*, and *PPP1R14B* differentiate a group of *circa* 5% of cases with short survival. This effect was independent of the main cytogenetic markers of adverse prognosis. Finally, this finding was reproduced in an independent retrospective cohort. We believe that this small gene expression pattern will be useful for CLL prognostication and its association with CLL response to novel drugs should be explored in the future.

## 1. Introduction

Chronic lymphocytic leukemia (CLL) is the most frequent lymphoproliferative syndrome in western populations, and it is characterized by its remarkable heterogeneous clinical evolution. In the molecular era of medicine, the discovery of new biomarkers is a central issue of disease prognostication. Recurrent cytogenetic aberrations, the *IGHV* hypermutation status, and, more recently, somatic mutations in driver genes such as *TP53*, *ATM*, *NOTCH1*, *SF3B1*, *MYD88*, and *BIRC3* have improved risk stratification of CLL patients [1–3].

The inherent continuous nature of gene expression supposes an opportunity to dissect heterogeneous tumor types into comprehensive molecular subclasses. Indeed, previous efforts have proven the usefulness of this approach in CLL prognostication. Rodríguez et al. reported a seven-gene signature correlated with *IGHV* mutation status that predicts time to treatment [4], whereas Herold et al. reported an 8-gene prognostic signature that predicted overall survival, but the predictability of this pattern was not superior to that of the combination of conventional FISH and *IGHV* mutation status [5].

Thus, we reasoned that the identification of new and small-sized patterns of gene expression associated with adverse survival and their dependency on the main cytogenomic factors of adverse prognosis may improve CLL prognostication.

## 2. Methods

We used two public databases of gene expression data in CLL patients in order to create a training and a validation cohort. The training cohort was composed of transcriptomic data from 450 CLL cases enrolled in the *International Cancer Genome Consortium* (data accessible in the *European Genome-phenome Archive*, accession code *EGAD00010000875*). Samples were collected and analyzed by the aforementioned consortium before initiation of any treatment. Overall survival was calculated as time from CLL diagnosis to time of death from any cause. Transcriptomic data were measured with Affymetrix HG-u219 microarrays. The *Robust Multichip Algorithm* (RMA) [6] was used to preprocess, normalize, and log2-transform expression data. For genes targeted by multiple probes, the median value was extracted. For each gene, we determined its individual clusterization capacity. The Mclust [7] algorithm was used in order to detect the 2 most likely patient clusters according to the expression of each gene (*Mclust function, parameter G* = 2). Briefly, the Mclust algorithm determines the most likely set of clusters according to geometric properties (distribution, volume, and shape). An expectation-maximization algorithm is used for maximum likelihood estimation, and the best model is selected according to Bayes information criteria. The association of each of these single-gene clusters with overall survival was calculated using cox regression. Thereafter, those genes whose clusterization was significantly associated with survival (*q*-value <0.05) were selected for multivariate clusterization using the same Mclust algorithm.

An independent cohort of 107 CLL samples was used for validation (accessible in the Gene Expression Omnibus, accession code GSE22762, array platform Affymetrix Human Genome U133 Plus 2.0 Array). This dataset was composed of samples from patients with newly diagnosed and preexisting CLL, a fraction of whom had been previously treated. Overall survival was calculated as the period of time from microarray analysis to death from any cause. Briefly, normalized gene expression estimates were extracted, median expression for multiprobe genes was calculated, and the array platform batch effect was adjusted using *Combat* [8]. Then, cluster prediction was performed with parameters estimated in the training cohort, and cox regression was used to verify the association of this clusterization with survival.

## 3. Results

**Figure 1:**
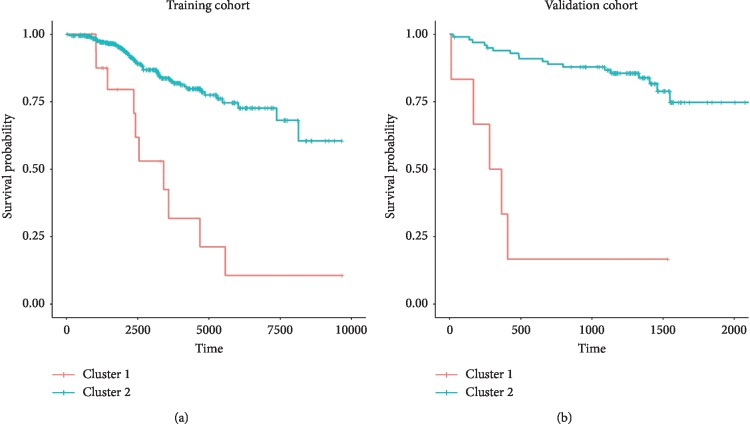
Kaplan–Meier plots representing the different evolution of CLL patients belonging to the two different clusters in the (a) training and (b) validation cohorts.

**Figure 2:**
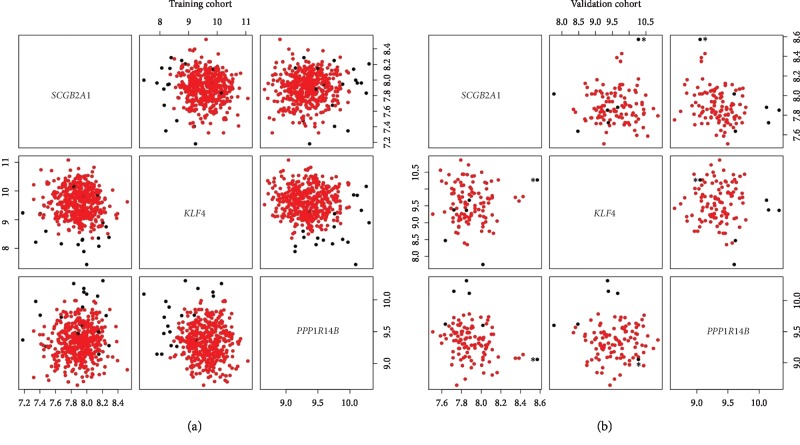
Scatterplot matrix representing the relationship of patients according to the expression of *SCGB2A1*, *KLF4*, and *PPP1R14B*. Separate plots are provided for the training (a) and validation (b) cohorts. Points are labeled according to the cluster assignation: black dots represent patients in the cluster of adverse prognosis and red dots represent the remaining group of patients. In (b), the asterisk indicates an event near the limit of both clusters (see text).

Three transcripts were able to individually clusterize patients in two groups with significantly different survival in the study cohort (Benjamini–Hochberg *q*-value <0.05) (Supplementary [Supplementary-material supplementary-material-1] and Supplementary [Supplementary-material supplementary-material-1]). These genes were *SCGB2A1*, *KLF4*, and *PPP1R14B*. A multivariate clusterization based on the three genes was created using Mclust. According to the BIC, the geometrical model rendering the optimal clusterization was diagonal, with varying volume and equal shape (*VEI* in Mclust argot). This clusterization was markedly associated with overall survival (cox regression *p*-value 4.31 × 10^−6^, hazard ratio 4.86, lower 95% confidence interval 2.48, upper 95% confidence interval 9.53; Figures 1(a) and 2(a)). The cluster of patients with adverse survival supposed 4.22% of the study cohort. The prognostic impact of this clusterization on survival was validated in an independent cohort (cox regression *p*-value 5.7 × 10^−6^, hazard ratio 10.79, lower 95% confidence interval 3.86, upper 95% confidence interval 30.17; Figures 1(b) and 2(b); Supplementary [Supplementary-material supplementary-material-1]). The cluster of patients with adverse survival represented 5.60% of the validation cohort. We could visually detect one case in validation cohort whose probability of belonging to the small cluster was 52.79% (indicated with an asterisk in Figure 2(b)). Discarding this event from the survival analysis did not significantly change the results: *p*-value 6.31 × 10^−6^; 95% HR: 0.03–0.26.

In order to assess the independence of our clusterization approach, we used data from Puente et al. [1] to analyze for potential confounders in the study cohort. The following covariates were included in the model: patient's age at diagnosis, Binet stage at diagnosis, *IGHV* mutation status, presence of *TP53* mutation or 17*p* deletion, *ATM* mutation or 11*q* deletion, *NOTCH1* mutation, *SF3B1* mutation, and *BIRC3* mutation. The association of the transcriptome clusterization remained significant independently of the effect of these adverse prognostic factors (cox regression *p*-value 8.95 × 10^−3^, hazard ratio 2.76). Since we could not get access to the status of these markers in the validation cohort, we could not reproduce this finding.

## 4. Discussion

In this paper, we present a new gene expression signature that identifies a group of CLL patients with shorter survival. The signature was composed of the following genes: *KLF4*, *SCGB2A1*, and *PPP1R14B*. *KLF4* belongs to the Kruppel family of transcription factors. *KLF4* has both growth suppressive and antiapoptotic functions since it can trigger cell-cycle arrest by inducing TP53-mediated expression of *CDKN1A* and it can also block apoptosis by inhibiting TP53 activity and suppressing *BAX* expression [9]. Less is known about *SCGB2A1* and *PPP1R14B*. *SCGB2A1* encodes a gene of the secretoglobin family. *SCGB2A1* is highly expressed in some tumor types [10], and it has been linked to adverse cancer prognosis in others [11]. *PPP1R14B* encodes a putative inhibitor of protein phosphatase 1, a pleiotropic enzyme that plays multiple functions in cellular growth, cell-cycle regulation, and apoptosis [12].

Patients from both cohorts were diagnosed and treated in the era of chemoimmunotherapy. A limitation of this analysis is that we ignore which treatment regimens (if any) were administered to each patient. Nevertheless, the remarkable strong association of the reported clusterization with overall survival in both the training and validation cohorts suggests a treatment-independent mechanism. It will be important to study the impact of new targeted drugs such as tyrosine kinase inhibitors or BCL2 antagonists in the survival of these CLL cases.

In conclusion, we report a 3-gene expression signature that identifies a subgroup ∼5% of CLL patients with short survival before the era of tyrosine kinase inhibitors. Furthermore, this clusterization in the training cohort was associated with adverse outcome independently of the most important cytogenomic factors.

## 5. Conclusions

A 3-gene expression signature characterizes a group of *circa* 5% of CLL patients with short survival. The prognostic impact of this signature was independent of the main cytogenomic markers of adverse prognosis at least in the study cohort. This small signature might be useful for future studies about disease prognostication and drug response in CLL.

## Data Availability

This study used public data accessible in the Gene Expression Omnibus and in the repository of the International Cancer Genome Consortium.

## Additional Points

A 3-gene expression signature identifies a subgroup of patients with chronic lymphocytic characterized by short survival. The classifier was independent of the main cytogenomic predictors of adverse prognosis in the study cohort.

## Supplementary Materials

Supplementary Materials Supplementary Figure 1: individual patient clusterization according to the expression of the three selected genes in the training cohort (red and blue bars). Black bars represent gene expression for each patient in the cohort. Supplementary Table 1: expression levels of *SCGB2A1*, *KLF4*, and *PPP1R14B*, survival data, and cluster membership of patients in the training cohort. Supplementary Table 2: expression levels of *SCGB2A1*, *KLF4*, and *PPP1R14B*, survival data, and cluster membership of patients in the validation cohort.
