# Supplementary figures and images for: A Three-Gene Expression Signature Identifies a Cluster of Patients with Short Survival in Chronic Lymphocytic Leukemia
Source: J Oncol. 2019 Nov 7;2019:9453539. doi: 10.1155/2019/9453539 (PMC6885206; doi:10.1155/2019/9453539)

**SUPPLEMENTARY FIGURE 1**


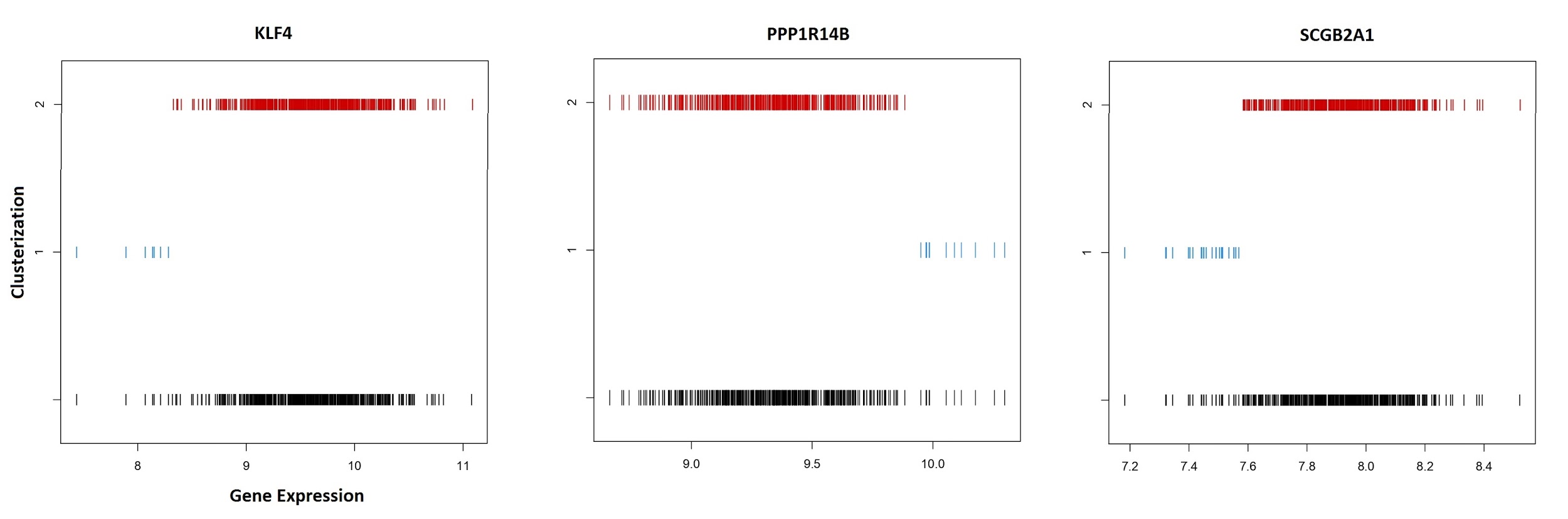

Supplement: Supplementary Materials — Supplementary Figure 1: individual patient clusterization according to the expression of the three selected genes in the training cohort (red and blue bars). Black bars represent gene expression for each patient in the cohort. Supplementary Table 1: expression levels of SCGB2A1, KLF4, and PPP1R14B, survival data, and cluster membership of patients in the training cohort. Supplementary Table 2: expression levels of SCGB2A1, KLF4, and PPP1R14B, survival data, and cluster membership of patients in the validation cohort. [file 9453539.f1.zip › 9453539.f1/SUPPLEMENTARY FIGURE 1_JO_2845135.docx]
